# The impact of cancer patient pathway on timing of radiotherapy and survival: a cohort study in glioblastoma patients

**DOI:** 10.1007/s11060-024-04709-z

**Published:** 2024-05-19

**Authors:** Hanne Blakstad, Eduardo Erasmo Mendoza Mireles, Kirsten Strømme Kierulf-Vieira, Divija Singireddy, Ibrahimu Mdala, Liv Cathrine Heggebø, Henriette Magelssen, Mette Sprauten, Tom Børge Johannesen, Henning Leske, Pitt Niehusmann, Karoline Skogen, Eirik Helseth, Kyrre Eeg Emblem, Einar O. Vik-Mo, Petter Brandal

**Affiliations:** 1 Department of Oncology, Oslo University Hospital, Oslo, Norway; 2 Institute of Clinical Medicine, University of Oslo, Oslo, Norway; 3 Department of Neurosurgery, Oslo University Hospital, Oslo, Norway; 4 Vilhelm Magnus Laboratory, Institute for Surgical Research, Oslo University Hospital, Oslo, Norway; 5 Institute of Health and Society, Faculty of Medicine, University of Oslo, Oslo, Norway; 6 Cancer Registry of Norway, Oslo, Norway; 7 Department of Pathology, Oslo University Hospital, Oslo, Norway; 8 Division of Cancer Medicine, Oslo University Hospital, Oslo, Norway; 9 Department of Radiology, Oslo University Hospital, Oslo, Norway; 10 Department of Physics and Computational Radiology, Division of Radiology and Nuclear Medicine, Oslo University Hospital, Oslo, Norway; 11 University of Oslo, Oslo, Norway; 12 Institute for Cancer Genetics and Informatics, Oslo University Hospital, Oslo, Norway

**Keywords:** Glioblastoma, Radiotherapy timing, Prognostic factors, Cancer patient pathway, Survival

## Abstract

**Purpose:**

Glioblastoma (GBM) is an aggressive brain tumor in which primary therapy is standardized and consists of surgery, radiotherapy (RT), and chemotherapy. However, the optimal time from surgery to start of RT is unknown. A high-grade glioma cancer patient pathway (CPP) was implemented in Norway in 2015 to avoid non-medical delays and regional disparity, and to optimize information flow to patients. This study investigated how CPP affected time to RT after surgery and overall survival.

**Methods:**

This study included consecutive GBM patients diagnosed in South-Eastern Norway Regional Health Authority from 2006 to 2019 and treated with RT. The pre CPP implementation group constituted patients diagnosed 2006–2014, and the post CPP implementation group constituted patients diagnosed 2016–2019. We evaluated timing of RT and survival in relation to CPP implementation.

**Results:**

A total of 1212 patients with GBM were included. CPP implementation was associated with significantly better outcomes (*p* < 0.001). Median overall survival was 12.9 months. The odds of receiving RT within four weeks after surgery were significantly higher post CPP implementation (*p* < 0.001). We found no difference in survival dependent on timing of RT below 4, 4–6 or more than 6 weeks (*p* = 0.349). Prognostic factors for better outcomes in adjusted analyses were female sex (*p* = 0.005), younger age (*p* < 0.001), solitary tumors (*p* = 0.008), gross total resection (*p* < 0.001), and higher RT dose (*p* < 0.001).

**Conclusion:**

CPP implementation significantly reduced time to start of postoperative RT. Survival was significantly longer in the period after the CPP implementation, however, timing of postoperative RT relative to time of surgery did not impact survival.

**Supplementary Information:**

The online version contains supplementary material available at 10.1007/s11060-024-04709-z.

## Introduction

Glioblastoma (GBM) is an aggressive and incurable primary brain tumor; median overall survival (mOS) in unselected patients is approximately 12 months [1, 2]. Treatment is multimodal; after surgery patients are scheduled for chemoradiotherapy. Patients 70 years or younger with good performance status receive radiotherapy (RT) to 60 Gy in 30 fractions. Concomitant and adjuvant temozolomide (TMZ) extends mOS about three months to 15 months [3, 4]. Hypofractionated RT to 40 Gy in 15 fractions with concomitant and adjuvant TMZ is the preferred standard treatment for elderly and/or younger relatively frail patients fit enough for anti-neoplastic treatment [5]. Tumor-treating fields is not reimbursed by the Norwegian public health care system and is thus not available as a treatment option in Norway [4].

**Fig. 1:**
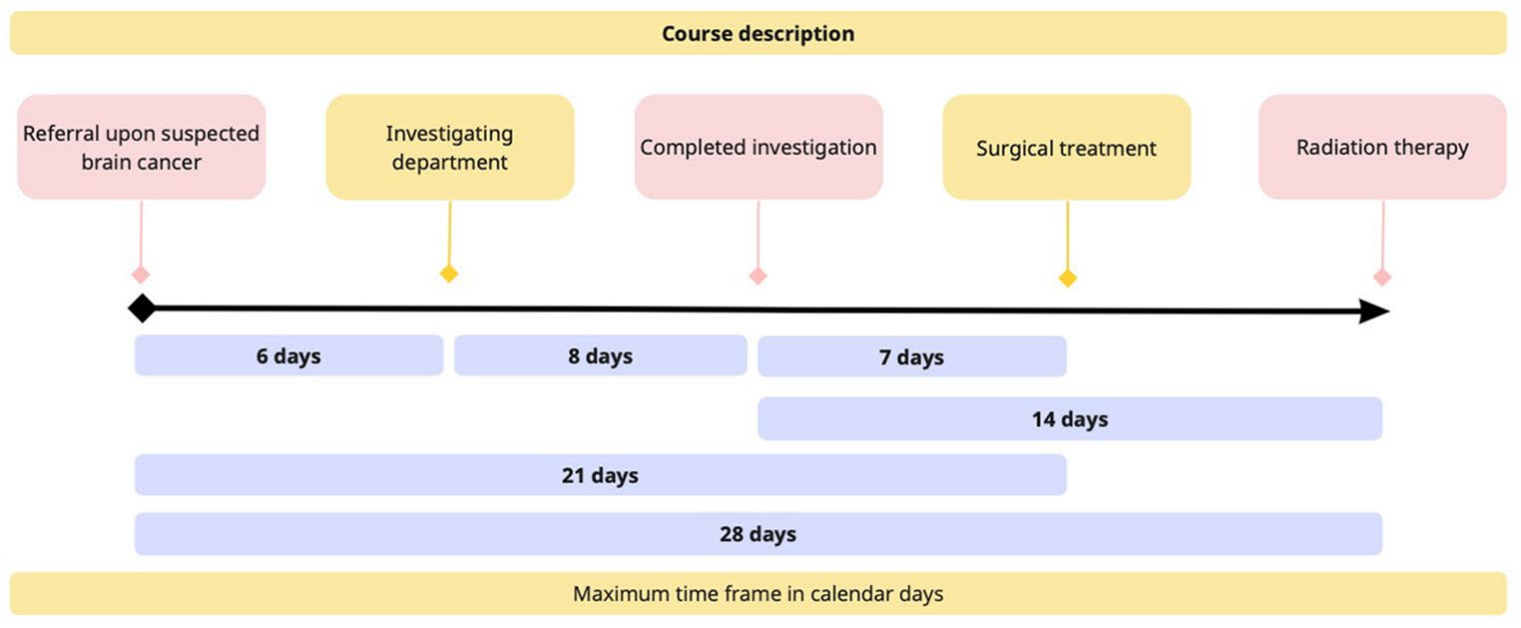
Pathway times in the national Norwegian brain cancer patient pathway. The figure was created at Miro.com

Over the last two decades, many countries have implemented initiatives to streamline cancer diagnosis, treatment, and patient care to reduce time from suspected cancer to treatment initiation [6–8]. The Norwegian Board of Health Supervision concluded in 2010 that non-medical delay in diagnosis and a lack of treatment continuity were significant challenges in cancer patient care [9]. Influenced by a similar Danish initiative, cancer patient pathways (CPPs) were implemented in Norway in 2015 to reduce non-medical delay and regional disparity [10]. The optimization of information and reduction of psychological distress for the patients was also emphasized. The CPPs describe a maximum waiting time from hospital referral to specialist visit, clinical decision, and treatment initiation, where each patient will be assigned a pathway coordinator to ensure timely and appropriate information. According to the progress timeline for the high-grade brain tumor-specific CPP, 70% of patients shall receive RT within three weeks from tumor-resective surgery or four weeks from the start of CPP if no tumor-resective surgery is performed (Fig. 1) [11].

The optimal timing of RT after surgery or biopsy in GBM patients is not well defined, although the rationale for prompt treatment seems intuitive given the aggressiveness of GBM. Some studies have reported improved outcomes with a delay in RT [12–14], whereas a few other studies found worse outcomes with a delay in RT [15, 16]. However, most studies found that timing of postoperative RT had no statistically significant impact on survival [17–22]. We conducted a retrospective cohort study including consecutive patients diagnosed with GBM from 2006 to 2019, aiming to investigate whether implementation of CPP impacted (1) time to RT after surgery and (2) overall survival.

## Materials and methods

### Patient cohort

We identified adult patients (> 18 years) diagnosed with GBM at Oslo University Hospital (OUH) from January 2006 to December 2019 in the Brain Tumor Registry at the Department of Neurosurgery. Neurosurgical Department of OUH covers the population of South-Eastern Norway Regional Health Authority, which has approximately 3.2 million inhabitants (55% of the Norwegian population). We included patients with histopathologically confirmed GBM according to the World Health Organization (WHO) classification of Tumors of the Central Nervous System (CNS) used at the time of diagnosis [23–25]. Also included were morphologically defined subgroups such as gliosarcoma, giant cell glioblastoma, and epitheloid glioblastoma. Patients who were lost to follow-up, had not received primary RT with photons, had no available information on RT start date, had received total brain RT, or TMZ monotherapy were excluded.

### Treatment

All patients underwent surgery at OUH’s neurosurgical department and went on to receive RT in South-Eastern Norway Regional Health Authority centers; most patients at OUH and some at Innlandet Hospital Trust and Sorlandet Hospital Trust.

### Data collection

We retrieved data from OUH’s internal quality registry and electronic patient journals. Collected data included patient demographics such as age and sex, tumor characteristics including location, and treatment at the time of primary diagnosis. Molecular genetic characteristics of methylated O^6^-methylguanine-DNA methyltransferase (*MGMT*) promoter methylation and isocitrate dehydrogenase (*IDH*) mutation status were not collected since the information was not available for most patients, or only immunohistochemistry had been performed to detect *IDH1* mutation. Extent of surgical resection was evaluated based on postoperative contrast-enhanced T1 MRI sequence and classified as gross total resection (GTR, no residual contrast-enhancing tumor), subtotal resection (STR, residual contrast-enhancing tumor), or biopsy. Although most patients received standard treatment with concomitant and adjuvant TMZ, all chemotherapy-relevant information was not fully available and, therefore, not included in this work.

### Defining time-periods for CPP

CPP was implemented gradually in 2015 according to national Norwegian guidelines. The CPPs define the maximum waiting time from hospital referral to specialist visit, clinical decision, and treatment initiation. Figure 1 shows the progress timeline of the high-grade brain tumor-specific CPP. A pathway coordinator ensures timely and appropriate information to the patients. We defined pre- and post CPP periods as 2006–2014 and 2016–2019, respectively. Start of RT was grouped as ≤ 4 weeks, 4.1-6.0 weeks, and > 6 weeks from surgery.

### Statistics

We used Stata version 17 (StataCorp LLC, Texas, USA) for statistical analyses. Overall survival was defined from date of primary surgery to death of any cause or censoring (January 25th, 2024) and was calculated using the Kaplan-Meier method with log-rank test. Cox-proportional hazard regression was used to determine predictors of survival for both univariate and multivariate models. *P*-values below 0.05 were considered statistically significant.

### Ethics

The Regional Committee for Medical and Research Ethics approved this study (592740), and they granted exemption from the need to obtain informed consent from all included patients.

## Results

### Patient and tumor characteristics

We identified 1410 patients diagnosed with GBM between 2006 and 2019. Of these, 198 patients were excluded due to loss to follow-up (*n* = 2), no RT (*n* = 149), RT with protons abroad (*n* = 1), TMZ monotherapy with or without later RT (*n* = 11), unknown start date of RT (*n* = 33), or whole brain RT (*n* = 2). For all patients identified, mOS was 11.7 months, and for excluded patients mOS was 2.0 months. A higher fraction of patients were excluded from the pre CPP than the post CPP group (16% vs. 11%). Of the 138 patients excluded in the pre CPP group, 107 patients (78%) did not receive RT and of these 54 (51%) were above 70 years. Of the 48 patients excluded from the post CPP group, 31 patients (65%) did not receive RT and the majority of these (*n* = 21, 68%) were above 70 years.

Inclusion criteria were met by 1212 patients (Table 1). Most patients were male (*n* = 724, 60%), and median age at diagnosis was 63 years (range 20–89 years). Median OS for included patients was 12.9 months, and 55% of patients were alive after one year, 21% after two years, and 6.1% after five years.

**Table 1:** Prognostic impact of patient, tumor, and treatment characteristics

Characteristics	Total	Median OS	Unadjusted analyses		Adjusted analyses	
	*N* (%)	Months	Hazard ratio (95% CI)	*P*-value	Hazard ratio (95% CI)	*P*-value
**All patients**	1212 (100)	12.9	**-**	-	-	-
Sex						
Male	724 (60)	12.8	1	-	1	-
Female	488 (40)	13.2	0.85 (0.75–0.95)	**0.005**	0.84 (0.75–0.95)	**0.005**
Age (years)						
<60	514 (42)	15.6	1	-	1	-
60–69	424 (35)	12.5	1.50 (1.32–1.71)	**< 0.001**	1.48 (1.30–1.70)	**< 0.001**
≥70	274 (23)	9.1	2.27 (1.95–2.64)	**< 0.001**	1.42 (1.16–1.74)	**0.001**
Tumor location						
Right side	608 (50)	12.8	1	-	1	-
Left side	565 (47)	13.1	0.95 (0.85–1.07)	0.398	1.01 (0.90–1.14)	0.866
Midline	12 (1.0)	13.7	0.97 (0.55–1.72)	0.919	0.82 (0.43–1.56)	0.547
Bilateral	27 (2.2)	9.1	1.67 (1.14–2.46)	**0.009**	1.25 (0.84–1.87)	0.279
Lobe						
Frontal	413 (34)	13.1	1	-	1	-
Parietal	206 (17)	13.6	1.02 (0.86–1.21)	0.810	1.07 (0.90–1.27)	0.459
Temporal	340 (28)	13.8	1.04 (0.90–1.20)	0.609	1.05 (0.90–1.22)	0.544
Occipital	93 (7.7)	13.4	1.29 (1.03–1.62)	**0.026**	1.28 (1.01–1.61)	**0.039**
Insula	20 (1.7)	10.8	1.54 (0.98–2.41)	0.061	1.54 (0.98–2.43)	0.060
Corpus callosum	29 (2.4)	10.4	1.53 (1.05–2.23)	**0.027**	1.34 (0.87–2.05)	0.179
Multifocal	111 (9.2)	9.4	1.59 (1.29–1.97)	**< 0.001**	1.35 (1.08–1.68)	**0.008**
Surgical resection						
GTR	339 (28)	16.7	1	-	1	-
STR	725 (60)	12.2	1.64 (1.44–1.88)	**< 0.001**	1.61 (1.41–1.85)	**< 0.001**
Biopsy	129 (11)	8.0	2.60 (2.11–3.19)	**< 0.001**	1.95 (1.56–2.44)	**< 0.001**
GTR by surgeon^a^	19 (1.6)	14.1	1.25 (0.79–1.99)	0.345	1.19 (0.74–1.90)	0.469
Radiotherapy						
54–60 Gy	960 (79)	14.8	1		1	
30–40.05 Gy	252 (21)	7.1	2.82 (2.45–3.26)	**< 0.001**	2.35 (1.92–2.86)	**< 0.001**

Significant *p*-values highlighted in bold

^a^Gross total resection deemed by neurosurgeon, but no available postoperative MRI

*Abbreviations* *OS* overall survival; *CI* confidence interval; *GTR* gross total resection; *STR* subtotal resection; *Gy* gray

### Treatment characteristics

A total of 339 patients (28%) underwent GTR, 725 patients (60%) underwent STR, and 129 patients (11%) had a biopsy performed (Table 1). The remaining 19 patients (1.6%) had GTR deemed by neurosurgeons and postoperative computer tomography scan, but there was no postoperative MRI to confirm it. The majority of patients (*n* = 960, 79%) had received standard RT (54–60 Gy), while the remaining (*n* = 252, 21%) had received hypofractionated RT (30–40.05 Gy). Median OS for the standard RT group was 14.8 months, and for the hypofractionated group 7.1 months.

The proportion of patients ≥ 70 years receiving standard RT was higher in the pre CPP group (*n* = 63/142, 44%) compared to the post CPP group (*n* = 21/114, 18%). Median OS for patients ≥ 70 years receiving standard RT was 17.7 months in the post CPP group compared to 10.5 months in the pre CPP group (HR 0.55, 95% CI 0.32–0.92; *p* = 0.022). The proportion of patients ≥ 70 years receiving hypofractionated RT was higher in the post CPP group (*n* = 93/114, 82%) compared to pre CPP group (*n* = 79/142, 56%). Median OS for patients ≥ 70 years receiving hypofractionated RT was 10.1 months in the post CPP group and 6.9 months in the pre CPP group (HR0.49, 95% CI 0.36–0.68; *p* < 0.001).

### Prognostic factors

Analyses are presented in Table 1. In adjusted analysis, statistically significant favorable prognostic factors were female sex, young age, solitary tumor, gross total resection, and high RT dose.

### Impact of cancer patient pathway on timing of RT

Patients diagnosed from 2006 to 2014 constituted the pre CPP group (*n* = 729, 60%), and patients diagnosed from 2016 to 2019 constituted the post CPP group (*n* = 396, 33%). The remaining 87 patients (7.2%) diagnosed in 2015 were excluded from this analysis (see Material and Methods). For the whole group, including patients diagnosed in 2015, median time from surgery to start of RT was 29 days. Median time from surgery to start of RT in the pre CPP group was 31 days, compared to 27 days in the post CPP group.

The fraction of patients that received RT ≤ 3 weeks from surgery, consistent with the national Norwegian CPP recommendation, was also higher in the post CPP group (*n* = 83/396, 21%) compared to the pre CPP group (*n* = 51/729, 7.0%) and was also statistically significant (OR 2.11, 95% CI 1.50–2.96; *p* < 0.001).

The fraction of patients that received RT ≤ 4 weeks from surgery was higher in the post CPP group (*n* = 258/396, 65%) compared to the pre CPP group (*n* = 285/729, 39%) (OR 1.94, 95% CI 1.49–2.51; *p* < 0.001). The fractions of patients who received RT 4.1-6.0 weeks and > 6 weeks from surgery were both higher pre CPP implementation when compared to post CPP implementation (OR 0.52, 95% CI 0.40–0.67; *p* < 0.001 and OR 0.11, 95% CI 0.06–0.21; *p* < 0.001, respectively).

### Impact of cancer patient pathway on survival

**Fig. 2:**
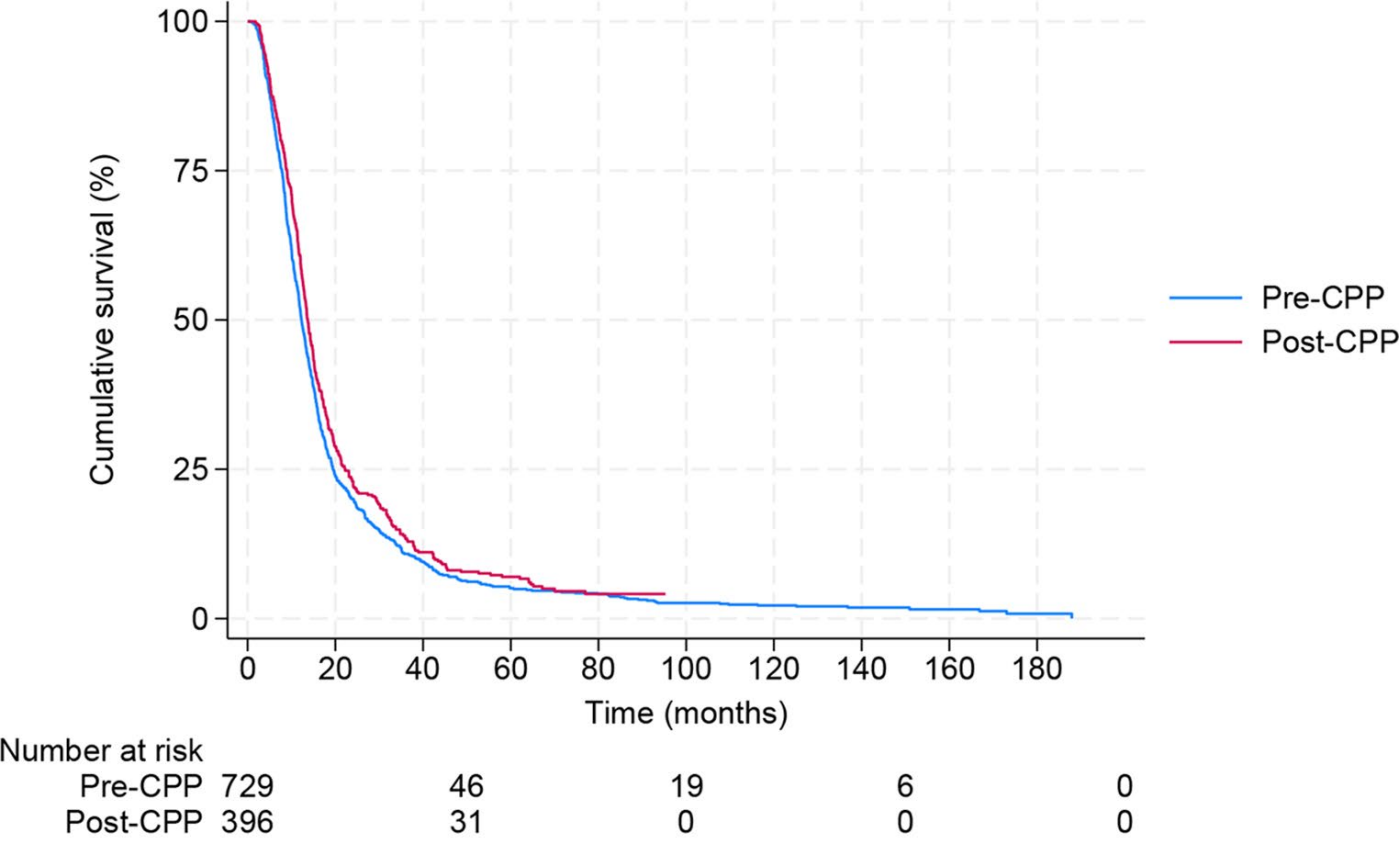
Survival before and after implementation of the national Norwegian brain cancer patient pathway. *Abbreviations*: CPP; cancer patient pathway

Median OS for the pre CPP group was 12.3 months, and for the post CPP group, 13.7 months (Fig. 2).

The difference in survival between the two groups was statistically significant unadjusted as well as adjusted for patient, tumor, and treatment factors; *p* = 0.039 and *p* < 0.001 (Table 2).

The subgroups that had significantly better outcomes post CPP compared to pre CPP implementation were patients 70 years and above(*p* = 0.001), patients with multifocal tumor (*p* = 0.005), patients with subtotal resected tumors (*p* = 0.040), and both the standard RT group (*p* = 0.009) and the hypofractionated RT group (*p* < 0.001) (Table 3).

**Table 2:** Prognostic impact of Cancer Patient Pathway implementation

Characteristics	Total	Median OS	Unadjusted analyses		Adjusted analyses	
	*N* (%)	Months	Hazard ratio (95% CI)	*P*-value	Hazard ratio (95% CI)	*P*-value
CPP						
Pre-CPP	729 (65)	12.3	1		1	
Post-CPP	396 (35)	13.7	0.88 (0.77–0.99)	**0.039**	0.73 (0.64–0.83)	**< 0.001**
Sex						
Male	668 (59)	12.8	1	-	1	-
Female	457 (41)	13.2	0.86 (0.76–0.97)	**0.014**	0.87 (0.77–0.98)	**0.023**
Age (years)						
<60	482 (43)	15.6	1	-	1	-
60–69	387 (34)	12.6	1.54 (1.34–1.77)	**< 0.001**	1.52 (1.32–1.74)	**< 0.001**
≥70	256 (23)	9.1	2.31 (1.97–2.70)	**< 0.001**	1.51 (1.22–1.85)	**< 0.001**
Tumor location						
Right side	562 (50)	13.0	1	-	1	-
Left side	528 (47)	12.9	0.97 (0.86–1.10)	0.579	1.04 (0.92–1.17)	0.574
Midline	12 (1.1)	13.7	0.97 (0.55–1.71)	0.911	0.93 (0.52–1.66)	0.814
Bilateral	23 (2.0)	9.1	1.67 (1.10–2.54)	**0.016**	1.24 (0.80–1.92)	0.327
Tumor focality						
Solitary	1025 (91)	13.3	1	-	1	-
Multifocal	100 (8.9)	9.2	1.41 (1.14–1.74)	**0.001**	1.27 (1.02–1.58)	**0.031**
Surgical resection						
GTR	329 (29)	16.4	1	-	1	-
STR	682 (61)	12.1	1.63 (1.42–1.87)	**< 0.001**	1.58 (1.38–1.82)	**< 0.001**
Biopsy	114 (10)	8.3	2.39 (1.92–2.96)	**< 0.001**	1.85 (1.46–2.33)	**< 0.001**
Radiotherapy						
54–60 Gy	889 (79)	14.8	1		1	
30–40.05 Gy	236 (21)	7.3	2.72 (2.34–3.15)	**< 0.001**	2.43 (1.97–2.99)	**< 0.001**

Significant *p*-values highlighted in bold

*AbbreviationsOS* overall survival; *CI* confidence interval; *GTR* gross total resection; *STR* subtotal resection; *Gy* gray

**Table 3:** Impact of cancer patient pathway on overall survival within groups

Characteristics	Pre-cancer patient pathway		Post-cancer patient pathway		Unadjusted analyses*	
	*N* (%)	Median OS (months)	*N* (%)	Median OS (months)	Hazard ratio (95% CI)	*P*-value
All patients**	729 (100)	12.3	396 (100)	13.7	0.88 (0.77–0.99)	**0.039**
Sex						
Male	435 (60)	12.1	233 (59)	13.5	0.90 (0.77–1.06)	0.216
Female	294 (40)	12.4	163 (41)	14.3	0.84 (0.69–1.03)	0.088
Age (years)						
<60	310 (43)	15.4	172 (43)	16.3	0.93 (0.76–1.13)	0.444
60–69	277 (38)	11.8	110 (28)	14.5	0.85 (0.68–1.06)	0.156
≥70	142 (19)	8.3	114 (29)	11.0	0.66 (0.51–0.85)	**0.001**
Multifocality						
Solitary	662 (91)	12.9	363 (92)	13.9	0.92 (0.81–1.05)	0.214
Multifocal	67 (9.2)	8.6	33 (8.3)	12.1	0.53 (0.34–0.82)	**0.005**
Surgical resection						
GTR***	195 (27)	15.8	134 (34)	17.3	0.93 (0.74–1.17)	0.555
STR	476 (65)	11.7	206 (52)	13.3	0.84 (0.71–0.99)	**0.040**
Biopsy	58 (8.0)	6.6	56 (14)	8.5	0.99 (0.68–1.44)	0.967
Radiotherapy						
54–60 Gy	612 (84)	14.0	277 (70)	15.7	0.82 (0.71–0.95)	**0.009**
30–40.05 Gy	117 (16)	6.0	119 (30)	9.7	0.51 (0.39–0.66)	**< 0.001**

Significant *p*-values highlighted in bold

*Pre-cancer patient pathway group used as reference

**All patients included in the pre- and post-cancer patient pathway groups

***Gross total resection deemed by surgeon, but no available postoperative MRI in *n* = 19

*Abbreviations* *OS* overall survival; *CI* confidence interval; *GTR* gross total resection; *STR* subtotal resection; *Gy* gray

### Impact of time to radiotherapy start on survival

All included patients (*n* = 1212) were part of this analysis. About half of all patients started RT ≤ 4 weeks of surgery (*n* = 605, 50%), followed by 4.1-6 weeks (*n* = 454, 37%), and > 6 weeks (*n* = 153, 13%) (Supplementary Table 1). The group with RT start ≤ 4 weeks from surgery (used as reference) had mOS of 13.3 months, compared to 12.3 months in both the 4.1-6 weeks group (HR 1.05, 95% CI 0.92–1.18; *p* = 0.482), and the > 6 weeks group (HR 1.08, 95% CI 0.90–1.29; *p* = 0.419). Survival was not statistically different between the groups (Supplementary Table 1). Likewise, there was no statistically significant survival difference by each increasing week interval from surgery to start of RT; HR 1.00, 95% CI 0.97–1.04; *p* = 0.790. Timing of postoperative RT start did not reach significance between the groups when adjusted for patient, tumor, and treatment factors (< 4 weeks used as reference): in the 4.1-6 week group; HR 1.04, 95% CI 0.92–1.18; *p* = 0.503 and in the > 6 week group HR 1.04, 95% CI 0.86–1.24; *p* = 0.702

## Discussion

We show that implementation of the national Norwegian brain CPP both significantly increased the fraction of patients starting RT ≤ 4 weeks postoperatively and positively impacted survival. However, we found that the timing of postoperative RT start relative to time of surgery did not impact survival

Better outcomes after CPP implementation are in accordance with a retrospective cohort study conducted in Denmark. The Danish study investigated if prognosis improved after CPP implementation for seven different cancer types and found significant improvement for all patients as a whole and for lung and gynecological cancers separately [26]. We also found that CPP implementation improved the fraction of patients who received RT ≤ 4 weeks from surgery. In Norway, a significant focus for CPP implementation was to ensure timely patient information, which is probably better accomplished with shorter waiting times from surgery to the start of RT.

In some previous reports, a delay in postoperative RT to > 4 weeks has been associated with better outcomes [12–14, 27, 28], while other studies have shown worse outcomes for patients with RT start > 4 weeks postoperatively [15, 16]. In a study with rat models, increased tissue damage was observed when the time interval from surgery to RT was shorter compared with longer [29]. In patients where only biopsy is performed (an unfavorable prognostic factor), they will usually be available for RT without waiting for postoperative reconstitution and thus receive early RT. Most previous studies, however, have found results comparable to ours; timing of postoperative RT had no statistically significant impact on survival [17–22]

Although we show that CPP impacted survival, we did not find a survival impact of postoperative RT timing. The CPPs also streamline time to diagnostics and surgery, and the timing of these variables is not included in this study. One should keep in mind that the introduction of CPP may advance the date of diagnosis to an earlier point in time and, therefore, introduce a lead-time bias [30, 31]. Another possible confounder is a more aggressive treatment approach in elderly patients related to the 2017 results showing that adding TMZ to hypofractionated RT resulted in longer survival compared to short-course RT alone [5]. In addition, the fraction of GBM patients operated with complete resection of contrast-enhancing tumors has increased over the years and has been shown to improve survival [32]. In our study, resection with GTR was higher in the post CPP period (34%) compared to the pre CPP period (27%)

As in most retrospective studies, our results may, in principle, be biased by patient selection. However, selection might be moderate as the population is geographically defined, large, complete, and consecutive - a major strength of this study. Although interesting, we do not have the *IDH* mutation and *MGMT* promoter methylation status, and for the GBM diagnosis, it is not important as all included patients were diagnosed and treated before the 2021 WHO classification [33]. A further limitation is the limited knowledge of concomitant and adjuvant temozolomide use; however, Norwegian patients uniformly received treatment with temozolomide since its introduction [3, 34]

As in most retrospective studies, our results may, in principle, be biased by patient selection. However, selection might be moderate as the population is geographically defined, large, complete, and consecutive - a major strength of this study. Although interesting, we do not have the *IDH* mutation and *MGMT* promoter methylation status, and for the GBM diagnosis, it is not important as all included patients were diagnosed and treated before the 2021 WHO classification [33]. A further limitation is the limited knowledge of concomitant and adjuvant temozolomide use; however, Norwegian patients uniformly received treatment with temozolomide since its introduction [3, 34]

In conclusion, the implementation of CPP impacted survival positively and undisputedly ensure that Norway’s publicly reimbursed national health care system is equally available to all citizens independently of patient, tumor, geographical factors, and socioeconomic status. However, RT start ≤ 4 weeks postoperatively did not show a survival benefit compared to more delayed RT. Molecular pathology is increasingly essential for diagnosing and subgrouping GBM [33] and may delay start of RT. The results from this study and numerous others [12–14, 17–22, 27, 28, 35, 36] support that a longer time from surgery to start of RT is safe, allowing a thorough diagnostic process to ensure optimal postoperative treatment and ease clinical interventional studies

### Electronic supplementary material

Below is the link to the electronic supplementary material.

Supplementary Material 1

## Data Availability

No datasets were generated or analysed during the current study.
