# Supplementary material for: The impact of cancer patient pathway on timing of radiotherapy and survival: a cohort study in glioblastoma patients
Source: J Neurooncol. 2024 May 19;169(1):137–45. doi: 10.1007/s11060-024-04709-z (PMC11269513; doi:10.1007/s11060-024-04709-z)
Supplement: Supplementary file 1 — Supplementary Material 1 [file 11060_2024_4709_MOESM1_ESM.docx]

|  | **<4 weeks** | | **4.1-6 weeks** | | | | **>6 weeks** | | | |
| --- | --- | --- | --- | --- | --- | --- | --- | --- | --- | --- |
| **Characteristics** | **N (%)** | **mOS** | **N (%)** | **mOS** | **Unadjusted analyses*** | | **N (%)** | **mOS** | **Unadjusted analyses*** | |
|  |  | **months** |  | **months** | **Hazard ratio**  **(95% CI)** | ***P*-value** |  | **months** | **Hazard ratio**  **(95% CI)** | ***P*-value** |
| **All patients** | 605 (100) | 13.3 | 454 (100) | 12.3 | 1.05 (0.92-1.18) | 0.482 | 153 (100) | 12.3 | 1.08 (0.90-1.29) | 0.419 |
| **Sex** |  |  |  |  |  |  |  |  |  |  |
| Male | 367 (61) | 13.5 | 258 (57) | 11.8 | 1.23 (1.04-1.44) | **0.013** | 99 (65) | 12.2 | 1.11 (0.89-1.39) | 0.370 |
| Female | 238 (39) | 13.0 | 196 (43) | 13.6 | 0.88 (0.73-1.07) | 0.212 | 54 (35) | 12.3 | 1.03 (0.76-1.40) | 0.824 |
| **Age (years)** |  |  |  |  |  |  |  |  |  |  |
| <60 | 270 (45) | 15.7 | 178 (39) | 15.6 | 0.95 (0.78-1.16) | 0.627 | 66 (43) | 15.6 | 0.94 (0.71-1.24) | 0.644 |
| 60-69 | 197 (33) | 13.5 | 179 (39) | 11.9 | 1.19 (0.97-1.46) | 0.093 | 48 (31) | 12.2 | 1.19 (0.87-1.64) | 0.280 |
| ≥70 | 138 (23) | 10.0 | 97 (21) | 8.9 | 0.97 (0.75-1.27) | 0.848 | 39 (26) | 7.3 | 1.36 (0.95-1.94) | 0.093 |
| **Tumor focality** |  |  |  |  |  |  |  |  |  |  |
| Solitary | 545 (90) | 13.9 | 412 (91) | 13.0 | 1.05 (0.92-1.20) | 0.446 | 144 (94) | 12.7 | 1.07 (0.89-1.29) | 0.473 |
| Multifocal | 60 (9.9) | 9.1 | 42 (9.2) | 9.4 | 1.02 (0.68-1.52) | 0.937 | 9 (5.9) | 9.8 | 1.56 (0.76-3.17) | 0.225 |
| **Surgical resection** |  |  |  |  |  |  |  |  |  |  |
| GTR** | 184 (30) | 17.5 | 144 (32) | 16.3 | 1.03 (0.82-1.29) | 0.817 | 30 (20) | 13.0 | 1.25 (0.84-1.87) | 0.277 |
| STR | 347 (57) | 12.8 | 264 (58) | 11.4 | 1.13 (0.96-1.33) | 0.135 | 114 (75) | 12.3 | 1.05 (0.85-1.30) | 0.640 |
| Biopsy | 74 (12) | 8.0 | 46 (10) | 7.2 | 0.84 (0.58-1.22) | 0.363 | 9 (5.9) | 9.1 | 0.56 (0.27-1.18) | 0.129 |
| **Radiotherapy** |  |  |  |  |  |  |  |  |  |  |
| 54-60 Gy | 470 (78) | 15.1 | 368 (81) | 13.7 | 1.08 (0.94-1.24) | 0.266 | 122 (80) | 14.0 | 1.09 (0.89-1.33) | 0.408 |
| 30-40.05 Gy | 135 (22) | 7.3 | 86 (19) | 6.8 | 1.01 (0.77-1.33) | 0.940 | 31 (20) | 6.5 | 1.16 (0.78-1.72) | 0.472 |
| **CPP***** |  |  |  |  |  |  |  |  |  |  |
| Pre-CPP | 285 (47) | 12.4 | 305 (67) | 12.0 | 1.05 (0.89-1.23) | 0.589 | 139 (91) | 12.7 | 1.00 (0.82-1.23) | 0.972 |
| Post-CPP | 258 (43) | 14.3 | 126 (28) | 13.3 | 1.02 (0.82-1.27) | 0.847 | 12 (7.8) | 7.5 | 1.17 (0.64-2.14) | 0.613 |

**Supplementary Table 1.** Impact of time from surgery to radiotherapy start on survival

*Radiotherapy start within 4 weeks used as reference

**Gross total resection deemed by surgeon, but no available postoperative MRI

******* Pre-CPP: 2006-2014, post-CPP: 2016-2019, excluded year 2015

Abbreviations: mOS; median overall survival, GTR, gross total resection; STR, subtotal resection; Gy, gray; CPP, cancer patient pathway
